# Stereotactic radioablation for recurrent or nearly incessant slow ventricular tachycardia treatment

**DOI:** 10.1093/europace/euae137

**Published:** 2024-05-23

**Authors:** Francesca De Lio, Marco Schiavone, Maria Elisabetta Mancini, Lorenzo Bianchini, Barbara Alicja Jereczek-Fossa, Claudio Tondo, Corrado Carbucicchio

**Affiliations:** Department of Clinical Electrophysiology & Cardiac Pacing, Centro Cardiologico Monzino, IRCCS, Via Carlo Parea 4, 20138 Milan, Italy; Department of Clinical Electrophysiology & Cardiac Pacing, Centro Cardiologico Monzino, IRCCS, Via Carlo Parea 4, 20138 Milan, Italy; Department of Periooperative Cardiology and Cardiovascular Imaging, Centro Cardiologico Monzino IRCCS, Milan, Italy; Department of Clinical Electrophysiology & Cardiac Pacing, Centro Cardiologico Monzino, IRCCS, Via Carlo Parea 4, 20138 Milan, Italy; Division of Radiation Oncology, IEO, European Institute of Oncology IRCCS, Milan, Italy; Department of Oncology and Hemato-Oncology, University of Milan, Milan, Italy; Department of Clinical Electrophysiology & Cardiac Pacing, Centro Cardiologico Monzino, IRCCS, Via Carlo Parea 4, 20138 Milan, Italy; Department of Biomedical, Surgical and Dental Sciences, University of Milan, Milan, Italy; Department of Clinical Electrophysiology & Cardiac Pacing, Centro Cardiologico Monzino, IRCCS, Via Carlo Parea 4, 20138 Milan, Italy

**Keywords:** Ventricular tachycardia, Slow ventricular tachycardia, Stereotactic radioablation, Radiofrequency catheter ablation, Arrhythmogenic substrate

Endo-epicardial radiofrequency catheter ablation (RFCA) stands as a well-established treatment for recurrent ventricular tachycardia (VT),^1,2^ reducing appropriate implantable cardioverter defibrillator (ICD) shocks and managing electrical storms (ES).^3^ Single-session high-dose stereotactic body radiation therapy (SBRT) represents a non-invasive alternative to RFCA, having demonstrated VT burden reduction.^4–7^ We designed a spontaneous, prospective, open-label study to validate SBRT in ICD carriers with recurrent refractory VTs and contraindications to RFCA or who have failed previous RFCAs. Currently, there is a scarcity of data regarding the effectiveness of SBRT in treating slow VTs below the ICD tachycardia detection interval (TDI). We report a sub-analysis from the STRA-MI-VT, regarding advanced heart failure (HF) patients with recurrent or nearly incessant VTs (NIVTs) below the ICD-TDI. Study methods have been reported elsewhere.^8^

**Figure 1:**
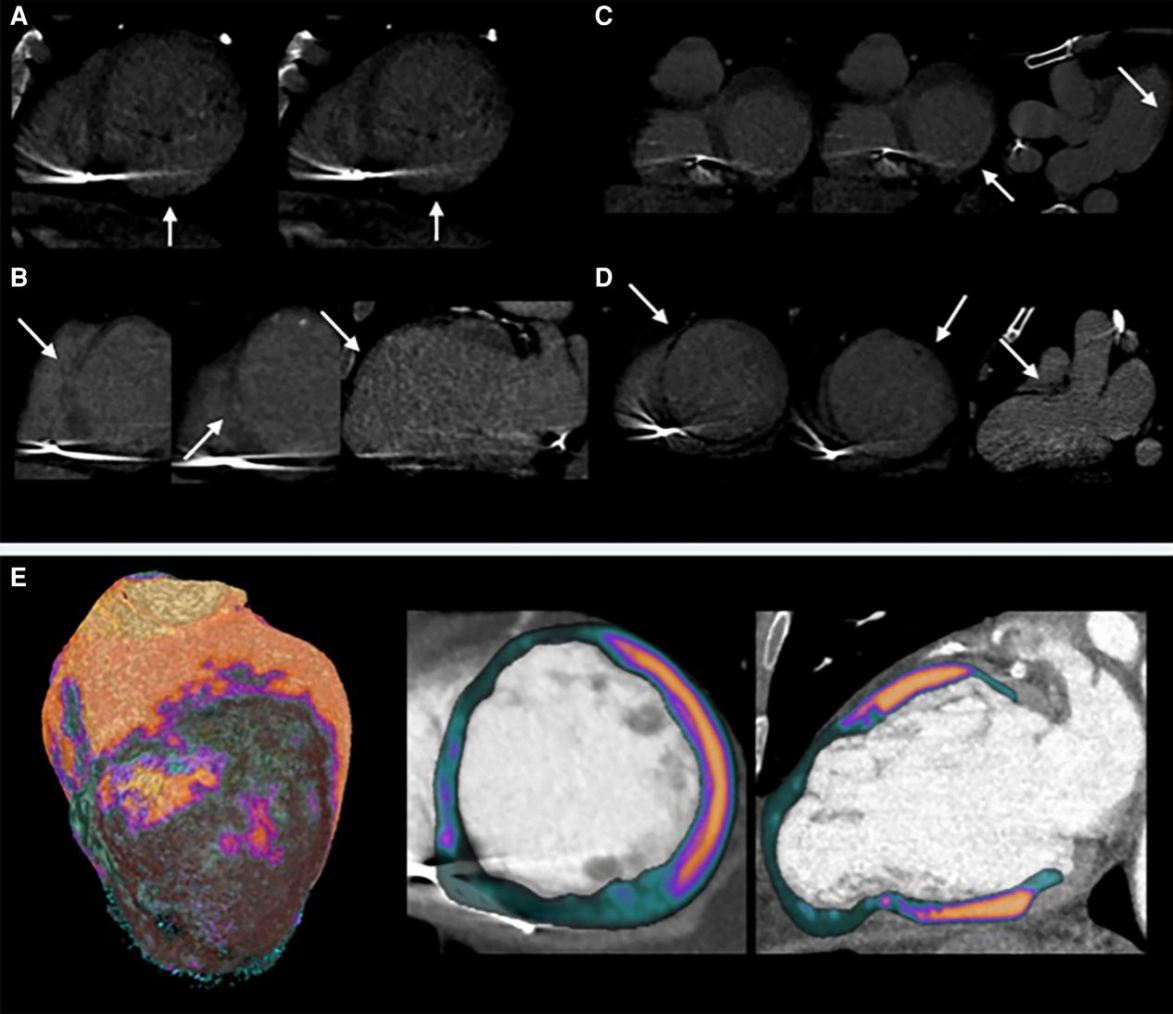
Late-iodine enhancement cardiac computed tomography showing: (*A*) Transmural late enhancement in the inferior wall in #Patient 1. (*B*) Transmural late enhancement in the mid-apical anterior wall and in the septum in #Patient 3. (*C*) Non-ischaemic late enhancement in the infero-lateral wall in #Patient 4. (*D*) Transmural late enhancement in all apical segments and in the mid antero-septal and antero-lateral wall in #Patient 5, Panel *E*. Arterial-phase computed tomography images of #Patient 5, reviewed with a resting myocardial perfusion assessment software, showing a thin-walled apical aneurism and an extensive area of hypoperfusion involving the mid-apical antero-septal and antero-lateral wall, periapical segments, and true apex.

The data that support the findings of this study are available from the corresponding author upon reasonable request. Among 15 patients enrolled in the STRA-MI-VT, five met the inclusion criteria for this sub-analysis. Median follow-up was 11 [3–13] months. Patients’ baseline characteristics and SBRT features have been summarized in *Table 1*. Imaging examples have been shown in *Figure 1*. All patients were males; mean age was 68 ± 5 years. Three patients were selected to receive SBRT for previous RFCA failures, while two patients were not deemed suitable for RFCA. All patients were on maximal antiarrhythmic drug (AAD) therapy [median AADs = 3 (1.5–3)]. Per-protocol, antiarrhythmic therapy was not modified during follow-up. Treatment characteristics have been summarized in *Table 1*. Mean clinical target volume was 40.5 ± 21.7 mL, resulting in a mean planning target volume of 180.2 ± 83.4 mL. Beam-on time was in all patients below 6 min. Mean *D*_95%_ and *V*_95%_ were 90.7 ± 10.1 and 93.6 ± 3.8%, respectively.

**Table 1:** Patients’ baseline and treatment characteristics

	Patient #1	Patient #2	Patient #3	Patient #4	Patient #5	Median [IQR]
Age (year)	72	72	61	67	69	69 [67–72]
Sex	M	M	M	M	M	
Underlying cardiomyopathy	ICM	NICM	ICM	NICM	ICM	
NYHA Class	III	III	III	I	II	
LVEF (%)	23.5	21.1	20.8	48.0	29.0	23.5 [21.1–29.0]
Device implanted	CRT-D	CRT-D	CRT-D	VVI ICD	DDD ICD	
Stage of chronic obstructive pulmonary disease (GOLD)	IV	III	II	I	I	
CKD stage	Severe	Severe	Mild-moderate	No	Mild	
BMI (kg/m^2^)	30.5	24.7	33.75	24.7	23.4	24.7 [24.7–30.7]
Thyroid function	Hyper-	Hypo-	no	no	Hypo-	
Atrial fibrillation	paroxysmal	permanent	no	no	Paroxysmal	
Arrhythmia presentation	VT, NIVT	VT, NIVT	VT, NIVT	VT, NIVT	VT, NIVT	
Prior cardiac surgery	yes	yes	no	no	No	
Clinical peculiarities	Mitra-clip	Mitro-aortic mechanic prosthesis, cardiac support device	Severe systemic arteriopathy	Previous cardiac tamponade/pericarditis	Ventricular thrombosis	
Ongoing AADs (*N*)	2	3	3	1	3	3.0 [1.5–3.0]
Previous VT catheter ablations: overall number (endo/epi)	3 (1 = endo-only, 1 = endo-epi, 1 = epi-only catheter ablation)	0	3 (3/0)	1 (1 = endo-epicardial catheter ablation)	0	
VT cycle length	420	460	440	430	500	440 [430–460]
Hospitalization rate (pre-treatment)^a^	7	2	4	2	4	4 [2–4]
Hospitalization rate (post-treatment)^a^	0	1	4	4	5	4 [1–4]
Tools used for target scar definition	Endo-epi EAM, CT, ECG	CT, ECGI, ECG	Endo EAM, CT, ECG	MRI, CT, ECG	CT, ECG	
Target scar location	Infero-postero-lateral	Basal perivalvular	Antero-septal, apex (LV aneurysm)	Sub-epicardial medio-basal infero-postero-lateral	Transmural apical and mid antero-lateral and antero-septal	
Clinical target volume (cm^3^)	43.7	16.04	53.35	21.4	67.86	43.7 [21.4–53.35]
Internal target volume (cm^3^)	115.9	54.4	145.5	72.4	204.5	116 [72–146]
Planning target volume (cm^3^)	198.3	88.1	239	99.8	275.6	198 [100–239]
*D*_95%_ (%)	94.9	96.2	95	72.7	94.7	94.9 [94.7–95]
*V*_95%_ (%)	94.8	97	95	87	94.1	94.8 [94.1–95]

AAD, antiarrhythmic drugs; ACBPG, aortocoronary bypass graft; BMI, body mass index; CKD, chronic kidney disease; CRT-D, cardiac resynchronization therapy defibrillator; CT, computed tomography; DDD ICD, dual-chamber implantable cardioverter defibrillator; EAM, electroanatomical mapping; ECG, electrocardiogram; ECGI, non-invasive electrocardiographic imaging; ES, electrical storm; ICD, implantable cardioverter defibrillator; ICM, ischaemic cardiomyopathy; IQR, interquartile range; LA, left atrium; LAD, left anterior descending artery; LAD, left anterior descending artery; LV, left ventricle; LVEF, left ventricular ejection fraction; NICM, non-ischaemic cardiomyopathy; NIVT, near-incessant ventricular tachycardia; NYHA, New York Heart Association; s.d., standard deviation; VT, ventricular tachycardia.

^a^The pre-treatment hospitalization rate was calculated over a period of time that was equal to the longest follow-up available for each patient.

After SBRT, we modified the ICD programming, changing the TDI so that clinical NIVTs could be recorded and, eventually, interrupted. A significant decrease in NIVTs was observed SBRT in all cases, with slow VTs completely resolving shortly after treatment. Two patients completed the 12-month follow-up period without any recurrence of slow VTs and without experiencing any treatment-related serious adverse events. Similarly, the last patient showed no slow VT recurrences during the 3-month follow-up period. Unfortunately, two patients died during follow-up, the first patient for worsening HF 11 months after SBRT, with no evidence of sustained slow VTs during the follow-up period. There was a progressive reduction in faster VTs, which fully disappeared 6 months after treatment. The second patient was discovered deceased at home during the third month after SBRT. Throughout the duration of the follow-up, slow VTs were eliminated following treatment, while some episodes of faster VTs persisted, although they decreased after SBRT. The SF-36-QoL questionnaire showed a slight improvement in physical functioning (26-to-48), role limitations due to physical health/emotional problems (33-to-55/22-to-50), health perception (43-to-48), and social functioning (46-to-79) from the pre-treatment to the last available follow-up.

To the best of our knowledge, this represents the first report assessing the SBRT for NIVTs. In 2022, Ninni *et al*.^7^ demonstrated the SBRT efficacy in addressing ES. Among the 17 patients analysed, five presented with ES associated with incessant VTs. Within this context, the timeframes of effectiveness varied, ranging from 1 to 7 weeks, mirroring our observations (0–6 weeks). The rationale behind this heterogeneity in response timing remains unclear, although it could be linked to the mechanisms of action inherent to SBRT.

Indeed, multiple cellular processes contribute to the mechanism of action of SBRT. Zhang *et al*. demonstrated, through electrophysiologic assessment of irradiated murine hearts, that SBRT may reactivate the Notch developmental signalling pathway, resulting in an up-regulation of sodium channel (Nav-1.5) expression. Moreover, Connexin-43, a constituent of gap junctions, undergoes up-regulation and lateralization 2 weeks following SBRT, persisting for at least 1 year.^9^ In human hearts instead, only the overexpression process of Nav-1.5 has been reported; this up-regulation may improve electrical conductivity, as evidenced by QRS and delta local activation time shortening.^10,11^ Although being speculative, this pro-conductive effect might help preventing unidirectional block, a pivotal event for re-entry initiation, particularly crucial in cases of NIVTs. Recently, Cha *et al*.^12^ reported that high-dose irradiation results in intercalated discs widening, intracellular cardiac sarcotubular system oedema, extracellular swelling, and diffuse mitochondrial damage leading to intracardiac conduction delay in rats. Instead, high dose of SBRT only rarely produced transmural fibrosis. These data suggest that SBRT early antiarrhythmic effects might be more related to cell-to-cell conduction disturbances and membrane potential alterations caused by inflammatory processes, rather than to fibrotic changes. Instead, the role of late-stage fibrosis in the homogenization of arrhythmogenic myocardial substrate, with subsequent disruption of the re-entry circuit, has limited available evidence, without solid evidence in humans. Thus, RT-induced fibrosis seems a dose-dependent phenomenon, with 25 Gy dose potentially being not sufficient to elicit myocardial fibrosis.^9^

Another plausible explanation for SBRT behavior in slow VTs may pertain to the size of the scar and the tachycardia isthmus. In such cases, the larger scars and VT isthmus related to the tachycardia cycle length may facilitate better-quality pre-procedural imaging necessary for defining the target volume and, conversely, greater precision and targeting in treatment delivery. Furthermore, while VT recurrences during follow-up may be frequent, it is noteworthy that recurrences are generally not observed within the planning target volume.^13^ Finally, although complications have been reported,^14^ no major adverse events clearly attributable to SBRT were found in our series. van der Ree *et al*.^4^ recently showed that SBRT is associated with worsening of valve function, whereas a significant change in left ventricular ejection fraction (LVEF) or development of coronary artery disease has not been observed. In our series, we did not observe worsening LV function {pre-SBRT LVEF median value 23.5 [interquartile range (IQR) 21.1–29.0] vs. post-SBRT LVEF median value 34.0 (IQR 26.0–35.0]}. One patient showed lung damage at the 3-month follow-up computed tomography scan, which was asymptomatic and without clinical impact (Grade 1 according to CTCAE document). The patient who was found dead 3 months after SBRT did not report any symptom prior to the event. While arrhythmic death was ruled out, the exact cause remained unknown, as no autopsy was performed.

Stereotactic body radiation therapy is linked to a notable reduction in slow-VT burden in the context of NIVT. Given its non-invasive nature, SBRT is a promising therapeutic tool for advanced HF patients who have exhausted all alternative treatment options.
